# Cæsarean Section; Mother and Child Saved

**Published:** 1854-09

**Authors:** José Angulo

**Affiliations:** Navia


Translations from the Spanish, made for the Examiner.

*Cæsarean section*; *mother and child saved.* By JOSÉ ANGULO of Navia. (Copied into "El Porvenir Medico," of Madrid, April 5, 1854, from "La Crónica de los hospitales.")

I was called, January 11th, 1854, to visit, (at a distance of three leagues from this city,) Juana Arduna, aged 37 years, native of Navia, in labor with her first child, and found her very miserable, lean, weak, and her height was scarcely three feet. She was in a small, dark room, supported by two women, and attended by a third as midwife. From the latter I learned that the labor had lasted already four days, and that the waters had been evacuated the preceding day, and the patient assured me that during pregnancy, with the exception of fluor albus, she had enjoyed good health. It was easy to perceive deformity of the pelvis, the right side of which formed a considerable projection, while the left was depressed; the symphysis pubis was compressed inwardly, and the antero-posterior diameter was scarcely two inches; I found difficulty in introducing the fingers into the strait of the pelvis, which compressed the fœtal head, strongly wedged in it: the orifice of the uterus was somewhat dilated, the waters had entirely escaped; the integuments of the fœtal cranium were swollen to such a degree that the fontanelles could not be detected. The pains succeeded each other with rapidity; the movements of the fœtus were enough to satisfy me that it was still alive. Upon the whole, I very much feared that death would terminate the case; embryotomy, a cruel operation which is dangerous for the mother, was contraindicated as long as signs of life were observed in the fœtus: the Cæsarean section, in my judgment, offered the best chance both for mother and child, and after explaining her condition and the hazards of the operation to the patient and her relatives, it was reluctantly acceded to, but the assistance of another physician was refused, on the ground that they were too poor to afford the additional fees, and besides, that their confidence in me was such, that whatever might be the result they never would reflect upon my honor and reputation. The patient had received, the day before, the sacrament of the Eucharist. At this point, she inquired whether she would suffer much pain during the operation. "No," I assured her, "scarcely will you feel it, wetting the corner of this pocket handkerchief with a liquid contained in this little bottle, and applying it to the nostrils; to which she replied, "she wished nothing of the kind, because she had courage enough to keep her lips closed while I operated."

The patient was placed horizontally on a table, and I made in the direction of the linea alba, an incision which extended from four lines below the umbilicus to the symphysis pubis; and then cutting the muscular parietes and introducing at the upper angle of the wound my left index finger to serve as a conductor, with a blunt pointed bistoury, I incised the peritoneum to the extent of the external wound. I had made scarcely half of this incision when an ounce of clear water escaped, which at first I believed proceeded from the bladder, but afterwards, saw that it did not. The opening of the integuments was about six inches long, and through it the uterus could he perceived, of a rubicund appearance. I made a small incision through its inferior part through which I introduced the extremity of my left index, and then cutting from below upwards, to the extent of five inches, I saw the fœtus presented the back and buttock. As the incision did not extend to the insertion of the placenta, no hæmorrhage supervened; I gently extracted the child from the womb. It was alive, and a female of moderate strength and constitution. The uterus contracted the moment the secundines were removed, and by aid of a gentle pressure, exit was given to a small quantity of blood which had entered the cavity of the abdómen, avoiding hernia of the intestines, by approximating the edges of the wound. The patient, who did not complain during the operation, was perfectly tranquil. The wound was dressed with a suture and adhesive straps, over which dry lint and compresses were applied, the whole sustained by a bandage around the body. As I could not visit the patient daily, I left instructions with the husband. I did not return to see her till after eight days; and I found her without fever, the lochia flowing well, and sufficient milk in the breast to suckle the child. At the end of thirteen days, only a small point remained to be cicatrized, and this was abandoned to nature: in a word, in a few days the patient was perfectly re-established, with plenty of milk, and the child in a very satisfactory condition.

NAVIA, Feb. 26, 1854.

